# p66Shc Deficiency in Chronic Lymphocytic Leukemia Promotes Chemokine Receptor Expression Through the ROS-Dependent Inhibition of NF-κB

**DOI:** 10.3389/fonc.2022.877495

**Published:** 2022-06-29

**Authors:** Vanessa Tatangelo, Gioia Boncompagni, Nagaja Capitani, Ludovica Lopresti, Noemi Manganaro, Federica Frezzato, Andrea Visentin, Livio Trentin, Cosima T. Baldari, Laura Patrussi

**Affiliations:** ^1^ Department of Life Sciences, University of Siena, Siena, Italy; ^2^ Hematology and Clinical Immunology Unit, Department of Medicine, University of Padua, Padua, Italy

**Keywords:** NF-kB, chronic lymphocytic leukemia (CLL), P66shc, ROS, chemokine receptor, CCR2, CCR7, CXCR3

## Abstract

The microenvironment of lymphoid organs is central to the pathogenesis of chronic lymphocytic leukemia (CLL). Within it, tumor cells find a favourable niche to escape immunosurveillance and acquire pro-survival signals. We have previously reported that a CLL-associated defect in the expression of the pro-apoptotic and pro-oxidant adaptor p66Shc leads to enhanced homing to and accumulation of leukemic cells in the lymphoid microenvironment. The p66Shc deficiency-related impairment in intracellular reactive oxygen species (ROS) production in CLL cells is causally associated to the enhanced expression of the chemokine receptors CCR2, CXCR3 and CCR7, that promote leukemic cell homing to both lymphoid and non-lymphoid organs, suggesting the implication of a ROS-modulated transcription factor(s). Here we show that the activity of the ROS-responsive p65 subunit of the transcription factor NF-κB was hampered in the CLL-derived cell line MEC-1 expressing a NF-κB-luciferase reporter following treatment with H_2_O_2_. Similar results were obtained when intracellular ROS were generated by expression of p66Shc, but not of a ROS-defective mutant, in MEC-1 cells. NF-κB activation was associated with increased expression of the chemokine receptors CCR2, CXCR3 and CCR7. Reconstitution of p66Shc in CLL cells normalized intracellular ROS and hampered NF-κB activation, which led to a decrease in the expression of these homing receptors. Our data provide direct evidence that the p66Shc-deficiency-related ROS depletion in CLL cells concurs to NF-κB hyperactivation and homing receptor overexpression, providing a mechanistic basis for the enhanced ability of these cells to accumulate in the pro-survival lymphoid niche.

## Introduction

Chronic lymphocytic leukemia (CLL) is characterized by the accumulation of long-lived mature CD5^+^ B cells in blood, bone marrow and secondary lymphoid organs (1). The extended leukemic cell survival is associated with impaired apoptosis, which in turn has been linked to a defect in the expression of the adaptor p66Shc and its transcription factor STAT4, typically observed in CLL cells (2, 3). CLL patients with the lowest levels of p66Shc expression have the worst disease presentation (2, 4). Moreover, leukemic cells isolated from CLL patients with aggressive disease secrete high amounts of interleukin (IL)-9, which stimulates stromal cells of lymphoid organs to secrete homing chemokines, thereby favouring their attraction to and retention in the pro-survival and protective lymphoid niche (5, 6). Enhanced expression of the homing receptors CCR2, CXCR3 and CCR7, paralleled by impaired expression of the egress receptor S1PR1 (7) also concurs to retain CLL cells in the lymphoid niche in both CLL patients (7–9) and Eμ-TCL1 mice (4), a well-established CLL mouse model (10). We recently demonstrated that the p66Shc defect of CLL cells participates in the dysregulated trafficking of CLL B cells. Indeed, both CCR2, CXCR3 and CCR7 are inversely correlated to p66Shc residual levels in CLL cells and can be normalized by restoring p66Shc, supporting the notion that p66Shc modulates the expression of these homing receptors.

p66Shc participates in signaling pathways linking oxidative stress to apoptosis (11, 12). In B lymphocytes, it negatively regulates cell survival by modulating the expression of several members of the Bcl-2 family of apoptosis-regulating proteins and inhibiting the activation of the pro-survival kinase Akt (2). Additionally, p66Shc promotes ROS production by interrupting the mitochondrial respiratory chain through cytochrome-c binding and oxidation, causing activation of the apoptotic cascade (13, 14). Intracellular ROS are consistently profoundly decreased in CLL cells as a result of the p66Shc defect (4), suggesting that a ROS-dependent transcription factor(s) might be involved in the enhanced expression of CCR2, CXCR3 and CCR7 in CLL cells.

The transcriptional activity of several classes of transcription factors is modulated by intracellular ROS (15). A prominent example is NF-κB, a family of transcription factors that bind DNA as homo- or heterodimers and whose activity is regulated by ROS at multiple levels of the signaling pathway leading to their activation (16). Moreover, ROS influence the DNA binding properties of the NF-κB proteins themselves. Oxidation of the p50 subunit on its DNA binding domain has been shown to prevent its DNA binding ability (17). On the other hand, a transient increase in intracellular ROS levels has been found to inhibit the phosphorylation of the p65 subunit (18). Interestingly, NF-κB has been reported to control the transcription of CCR2, CXCR3 and CCR7 in cancer cells (19–21).

Enhanced NF-κB activation contributes to the pathophysiology of CLL, although the underpinning mechanism remains largely to be elucidated. Enhanced BCR (22) and TLR (23) signaling, as well as interaction with stromal cells (24), have been proposed as the main source of aberrant NF-κB activation in CLL cells (25). Moreover, Vaisitti and colleagues demonstrated that the transcriptional activity of the p65 subunit of NF-κB is affected in CLL cells (24). However, the specific role of intracellular ROS in NF-κB activation in CLL cells has not been dissected to date. Here, we have addressed the outcome of the p66Shc deficiency-related impairment in intracellular ROS production on NF-κB activation in CLL B cells. We show that low intracellular ROS in CLL cells lead to enhanced activation of the p65 subunit of NF-κB and increased expression of the chemokine receptors CCR2, CXCR3 and CCR7, a defect that was reversed by restoring p66Shc expression. Our data provide direct evidence that ROS depletion caused by p66Shc deficiency concurs to NF-κB hyperactivation in CLL.

## Methods

### Cell Lines, Patients, Healthy Donors and Reagents

Peripheral blood (PB) samples were collected from 29 treatment-naive CLL patients. Diagnosis of CLL was made according to international workshop on CLL (iwCLL) 2008 criteria (26, 27). The main clinical features of CLL patients used in this study are listed in **Supplementary Table 1**. B cells from 14 buffy coats were used as healthy population controls. B cells were purified by negative selection using RosetteSep B-cell enrichment Cocktail (StemCell Technologies, Vancouver, Canada) followed by density gradient centrifugation on Lympholite (Cedarlane Laboratories, The Netherlands). Stable transfectants generated using the CLL-derived B-cell line MEC-1 (28) and expressing human full-length p66Shc or the p66ShcQQ mutant were previously described (7). H_2_O_2_ (#H1009), Lipopolysaccharide (LPS, #LPS25), DMSO (#102952), phorbol 12-Myristate 13-Acetate (PMA, #19-144), and A23187 (#C5722) were purchased from Merck. IT-901 was from R&D Systems (#5846).

### Transfections and Luciferase Assays

B cells purified from healthy donors and CLL patients were transiently co-transfected with 3 μg NF-κB-luciferase reporter vector and 3 μg pcDNA3 or p66Shc-encoding pcDNA3 or p66ShcQQ pBabe vectors/sample using the Human B-cell Nucleofector Kit (Amaxa Biosystems, Cologne, Germany) as described (4). MEC-1 transfectants were transfected with 5 μg NF-AT-luciferase or NF-κB-luciferase reporter vectors using a modification of the DEAE/dextran procedure (MEC cells), as described (29). 24 hours post-transfection, 1×10^6^ cells were resuspended in 1 ml RPMI w/o BCS and treated with either H_2_O_2_ at the indicated concentration or a combination of PMA (100 ng/ml) and A23187 (500 ng/ml) as described previously (30). Cells were collected 6 hours after activation and processed for luciferase assays as described previously (31) using the fluoroscan microplate reader Tecan Infinity Pro F200. The luciferase activity, normalized to the total protein amount quantified by IMarkTM Plate Reader (BioRad) using the Quantum Protein assay kit (Euroclone, #EMP14250), was expressed as Relative Luciferase Units (RLU). All samples were performed in duplicate. The transfection efficiency of the NF-κB-luciferase reporter and p66Shc-encoding vectors was assessed by qRT-PCR of p66Shc and flow cytometric analysis of the percentage of luciferase^+^ cells using mouse anti-firefly luciferase antibodies (Novus Biotechnology, #NB600-307ss) (**Supplementary Figure 1**).

### RNA Purification and RT-PCR

RNA was extracted and retrotranscribed as described (32). Real-time PCR was performed in triplicate on 96-well optical PCR plates (Sarstedt) using SSo FastTM EvaGreenR SuperMix and a CFX96 Real-Time system (Bio-Rad Laboratories). Results were processed and analyzed as described (32). Transcript levels were normalized to HPRT1. Primers used for amplification are listed in **Supplementary Table 2**.

### Cell Treatments, Apoptosis, ROS Measurement, Flow Cytometry and Immunoblot

Cells were treated with IT-901, LPS, or H_2_O_2_ at the indicated concentrations. Control samples were treated with DMSO. Surface expression of chemokine receptors was assessed after 24 hours by flow cytometry on cells labeled with anti-CCR2 (Thermo Fisher Scientific, #PA5-23043, 20 µg/ml), anti-CXCR3 (Thermo Fisher Scientific, #702228, 10 µg/ml) or anti-CCR7 (R&D Systems, #FAB197F) antibodies. For CCR2 and CXCR3 stainings, Alexa Fluor-488 anti-rabbit secondary antibodies (Thermo Fisher Scientific, #A11008) were used. Phospho-NF-κB was quantitated after 30 minutes by flow cytometry in cells fixed and permeabilized using Fixation (Biolegend, #420801) and Permeabilization (Biolegend, #421002) buffers and stained with anti-human phospho-NF-κB p65 (Ser536) (Cell Signaling Technology, #3031) and Alexa Fluor-488 anti-rabbit secondary antibodies. Intracellular ROS were measured by flow cytometry in cells labeled for 30 min at 37°C with 5 μM CM-H_2_DCFDA (4). Cell viability was measured by flow cytometric analysis of 1×10^6^ cells co-stained with FITC-labeled Annexin V (BioLegend, #640906) and Propidium iodide (PI, 20 µg/mL, Biotium, #40017) stained cells. Flow cytometry was performed using Guava Millipore cytometer as described (8).

Cells (5×10^6^ cells/sample) were lysed in 1% (v/v) Triton X-100 in 20 mM Tris-HCl pH 8, 150 mM NaCl, in the presence of a cocktail of protease inhibitors (Calbiochem, #539134) and 0.2 mg/ml Na orthovanadate (Merck, #S6508), resolved by SDS-PAGE (Life Technologies, #NW04120BOX) and transferred to nitrocellulose (GE Healthcare, #9004-70-0) as previously described (32). Immunoblots were carried out using rabbit anti-human phospho-NF-κB p65 (Ser536) (Cell Signaling, #3033), rabbit anti-Shc (Merck Millipore, #06-203) and anti-actin (Millipore, #MAB1501) primary antibodies. Secondary peroxidase-labeled anti-mouse (#115-035-146) and anti-rabbit (#111-035-003) antibodies were from Jackson Immuno-Research. Labeled antibodies were detected using ECL kit (SuperSignal^®^ West Pico Chemiluminescent Substrate, Thermo Scientific), and immunoblots were digitally acquired and analyzed using Alliance Q9-Atom chemiluminescence imaging system (Uvitec).

### Chromatin Immunoprecipitation

ChIP assays for the analysis of p65 NF-κB binding to the promoters of CXCR3, CCR2 and CCR7 was carried out using MAGnify Chromatin Immunoprecipitation System (Thermo Fisher Scientific, #492024). 1 × 10^6^ MEC-1 cells either untreated or treated for 1 hour with 1 μM IT-901 were crosslinked with 1% formaldehyde for 10 min at room temperature. Cells were lysed and sonicated 5 times for 10 sec to obtain average DNA fragment sizes of 300-500 bases. Immunoprecipitations were carried out using 5 μg of either anti-p65 NF-κB (Thermo Fisher Scientific, #510500) or control rabbit IgG. The immunoprecipitated DNA fragments were quantitated by qRT-PCR as described above. The primer sets used for the analyses are listed in **Supplementary Table 2**.

### Statistical Analyses

Two-way ANOVA with *post-hoc* Tukey were used for experiments where multiple groups were compared. Mann-Whitney rank-sum and paired t-tests were performed to determine the significance of the differences between two groups. One-sample Wilcoxon tests were used where samples were compared to a known standard value. Statistical analyses were performed using GraphPad Software (La Jolla, CA). P values <0.05 were considered significant.

### Study Approval

Written informed consent was received from CLL patients and healthy donors prior to inclusion in the study according to the Declaration of Helsinki. Experiments were approved by the local Ethics Committee.

## Results

### NF-κB Is Regulated by ROS and Controls the Expression of CCR2, CXCR3 and CCR7 in CLL Cells

We have previously reported that CLL B cells show a decrease in ROS production compared to their normal counterparts, that is associated with the enhanced transcription of genes encoding the homing receptors CCR2, CXCR3 and CCR7 (4, 7). We asked whether a shared ROS-modulated transcription factor is implicated in their transcriptional regulation. We performed an *in-silico* analysis using the JASPAR software on the *ccr2*, *cxcr3* and *ccr7* loci, focusing on ~2 kb regions upstream of the respective transcription start sites, and searched for putative binding sites for transcription factors known to be active in lymphocytes and that have been reported to be regulated by ROS. Among immune-related transcription factor families, we selected FOXO, ELK, NF-AT, the p50 (NF-κB1) and p65 (Rel A) subunits of NF-κB, NRF and the AP1 dimer JUN : FOS based on their known ROS sensitivity (15, 33–38) (**Table 1**). Putative binding sites for members of the selected families of transcription factors were found in the ~2 kb DNA regions upstream of the coding sequence of all three analyzed genes. Among them, we further restricted our analysis to transcription factors whose binding score exceeded 8.0, which led to the identification of NF-ATC2 and NF-κB as the best candidates (**Table 1**; **Supplementary Table 3**).

**Table 1:** List of transcription factors whose putative interaction sites were found in the promoters of the indicated genes.

Gene	Matrix ID	Matrix name	Max Score	N. putative sites
***ccr2***	MA0076.2	ELK4	7.8	8
	MA0099.1	JUN::FOS	11.8	12
	MA0105.1	NFĸB1	8.0	2
	MA0107.1	Rel A	8.6	4
	MA0152.1	NFATC2	11.4	24
	MA0157.1	FOXO3	5.3	17
	MA0506.1	NRF1	2.2	1
***cxcr3***	MA0076.2	ELK4	12.0	5
	MA0099.2	JUN::FOS	7.8	8
	MA0105.1	NFĸB1	10.6	10
	MA0107.1	Rel A	10.9	7
	MA0152.1	NFATC2	11.4	12
	MA0157.1	FOXO3	7.8	39
***ccr7***	MA0076.2	ELK4	11.7	5
	MA0099.1	JUN::FOS	10.7	26
	MA0105.1	NFĸB1	9.0	4
	MA0107.1	Rel A	9.1	2
	MA0152.1	NFATC2	11.3	15
	MA0480.1	FOXO1	11.9	13
	MA0157.2	FOXO3	7.4	18

**Figure 1:**
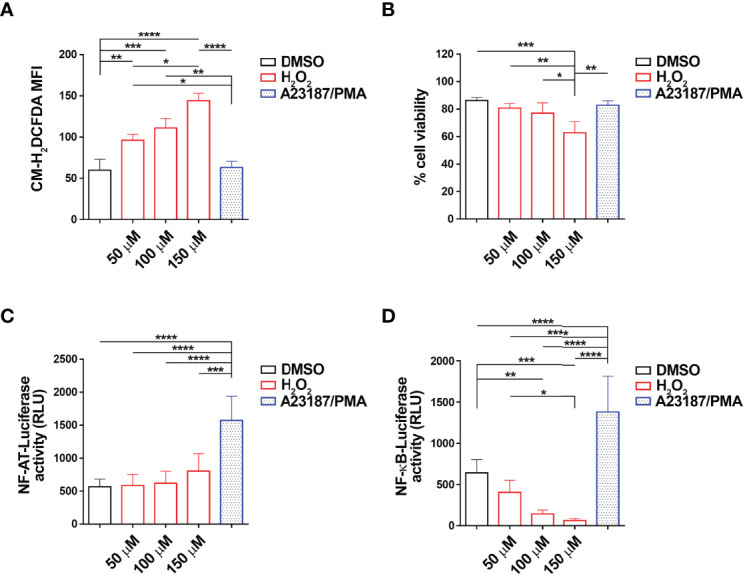
H_2_O_2_ suppresses NF-κB activity in MEC-1 cells. **(A–D)**. Quantification of the luciferase activity in MEC-1 cells transfected with either NF-AT-luciferase **(C)** or NF-κB-luciferase **(D)** reporter constructs and then treated for 6 h with H_2_O_2_ at the indicated concentrations. Data are expressed as relative luciferase units (RLU). A combination of A23187 and PMA was used as positive control (n independent experiments = 4). A mix of cells transfected with the two constructs and treated as above were used for the flow cytometric analysis of ROS intracellular content in cells stained with 5 μM CM-H_2_DCFDA **(A)** and of cell viability, calculated as the percentage of Annexin V^-^/PI^-^ cells **(B)** (n independent experiments = 4). Mean ± SD. Anova two-way test, Multiple Comparison. p ≤ 0.0001, ****; p ≤ 0.001, ***; p ≤ 0.01, **; p ≤ 0.05, *.

To address the potential role of ROS in the regulation of the transcriptional activity of NF-ATC2 and NF-κB, we transiently transfected the CLL-derived human B cell line MEC-1 (28) with constructs encoding firefly luciferase under the control of a trimer of the either a NF-AT or a NF-κB binding site. After 24 h, transfectants were treated for 6 h with 50, 100 or 150 μM H_2_O_2_ as exogenous source of ROS, and luciferase activity was measured. Intracellular ROS content and cell viability of the MEC-1 transfectants were quantified by flow cytometric analysis of the percentage of CM-H_2_DCFDA^+^ and Annexin V^-^/PI^-^ cells, respectively (**Figures 1A**, **B**). H_2_O_2_ significantly enhanced intracellular ROS in MEC-1 transfectants in a dose-dependent manner (**Figure 1A**) with minor effects on cell viability, with the exception of the highest H_2_O_2_ concentration which significantly enhanced cell death (**Figure 1B**). Interestingly, while the NF-AT-dependent luciferase activity was not affected, the NF-κB-dependent luciferase activity was strongly suppressed by H_2_O_2_ treatment in a dose-dependent manner (**Figures 1C, D**), indicating that NF-κB activity is reduced by intracellular ROS in B lymphocytes.

**Figure 2:**
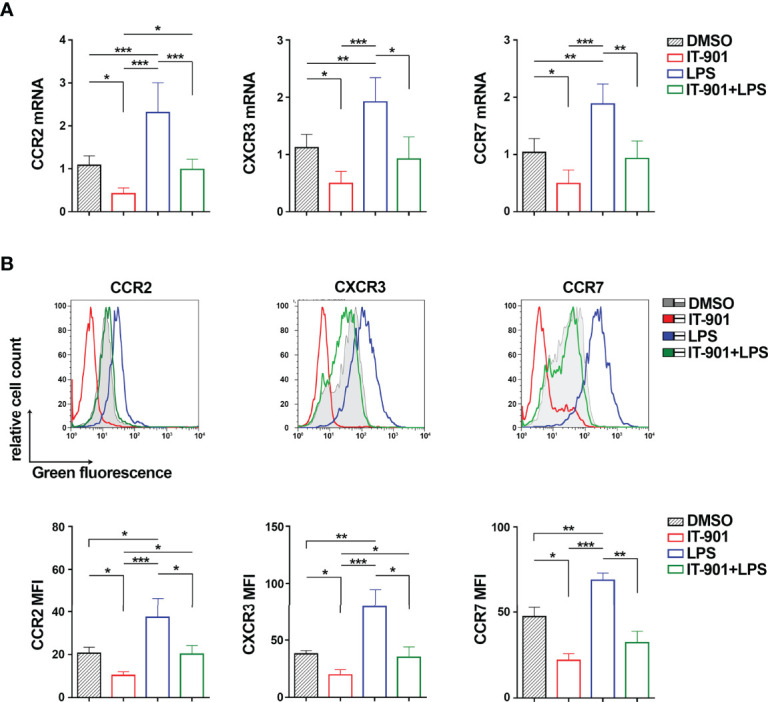
NF-κB coordinately controls CCR2, CXCR3 and CCR7 expression in MEC-1 cells. Quantitative RT-PCR **(A)** and flow cytometric **(B)** analysis of CCR2, CXCR3 and CCR7 mRNA **(A)** and protein **(B)** in MEC-1cells treated with 1 μM IT-901 or 10 μM LPS or the combination of both for 24 h at 37°C. The relative gene transcript abundance was determined on triplicate samples using the ddCt method and normalized to HPRT1 (n independent experiments = 4). Representative flow cytometric plots are shown. Mean ± SD. Anova two-way test, Multiple Comparison. p ≤ 0.001, ***; p ≤ 0.01, **; p ≤ 0.05, *.

We next assessed whether NF-κB coordinately controls the expression of CCR2, CXCR3 and CCR7. MEC-1 cells were treated for 24 hours with either IT-901, a NF-κB inhibitor that has been proven efficient in inhibiting NF-κB in CLL cells (24), or LPS, a known activator of the NF-κB pathway (39), or a combination of both. mRNA and surface expression levels of CCR2, CXCR3 and CCR7 were quantified by qRT-PCR and flow cytometry, respectively. Treatment with IT-901 significantly reduced both mRNA and surface levels of CCR2, CXCR3 and CCR7 (**Figure 2**; **Supplementary Figure 2**). Conversely, treatment of MEC-1 cells with LPS enhanced the surface expression of CCR2, CXCR3 and CCR7 (**Figure 2**; **Supplementary Figure 2**). Interestingly, IT-901 abrogated the LPS-dependent enhancement in expression of the three chemokine receptors (**Figure 2**) without significantly affecting cell viability (**Supplementary Figure 3A**).

**Figure 3:**
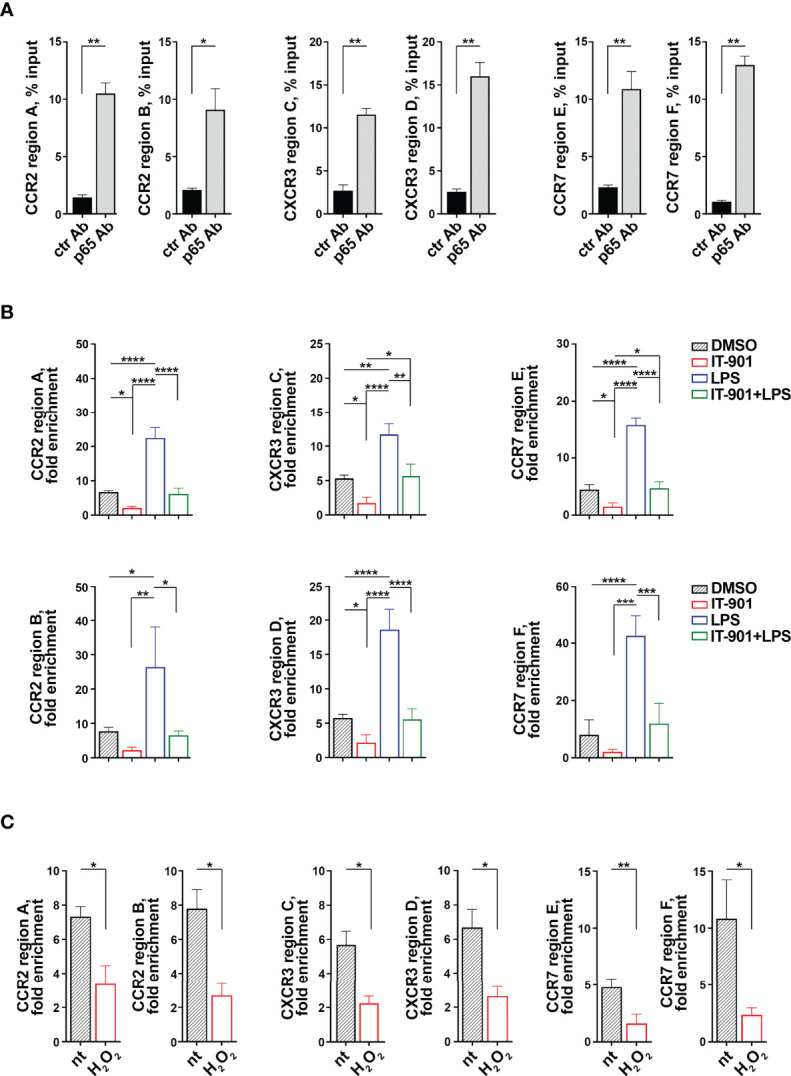
The p65 subunit of NF-κB binds to the *ccr2, cxcr3 and ccr7* promoters in MEC-1 cells. **(A)** ChIP assays of nuclear extracts of MEC-1 cells using either anti-NF-κB p65 (p65 Ab) or control unspecific Rabbit IgG (ctr Ab) antibodies. Selected regions of the *ccr2, cxcr3 and ccr7* promoters containing putative binding sites for the p65 subunit of NF-κB were amplified by qRT-PCR. Data are presented as percentage of input DNA (n = 4). **(B)** ChIP assays of nuclear extracts of MEC-1 cells treated either with DMSO or with 1 μM IT-901 or 10 μM LPS or the combination of both for 30 min at 37°C and then subjected to qRT-PCR as described above. Data are presented as fold enrichment (percentage of input DNA of p65-Ab IP samples vs ctr Ab samples) (n = 3). **(C)** ChIP assays of nuclear extracts of MEC-1 cells either untreated or treated with 100 μM H_2_O_2_ for 30 min at 37°C and then subjected to qRT-PCR as described above. Data are presented as fold enrichment (percentage of input DNA of p65-Ab IP samples vs ctr Ab samples) (n = 3). The relative gene transcript abundance was determined on triplicate samples using the ddCt method. Mean ± SD. **(B)** Anova two-way test, Multiple Comparison. **(A, C)** Paired t test. p ≤ 0.0001, ****; p ≤ 0.001, ***; p ≤ 0.01, **; p ≤ 0.05, *.

Since the transcriptional activity of the p65 subunit of NF-κB is affected in CLL cells (24), we performed Chromatin Immunoprecipitation (ChIP) assays on MEC-1 cells to assess whether p65 binds to the *ccr2*, *cxcr3* and *ccr7* promoters (**Table 1**). Two sets of primers were designed on each gene promoter to amplify DNA regions containing the putative p65 binding sites with the highest binding scores (**Table 1**) (a detailed list of the sequences amplified by qRT-PCR is shown in **Supplementary Table 4**). P65 binding was evaluated by ChIP assays using anti-p65 IgG or control non-specific IgG antibodies followed by qRT-PCR of the regions of interest and normalized to the input. A statistically significant binding of p65 to the *ccr2*, *cxcr3* and *ccr7* promoters was observed when ChIP assays were carried out using anti-p65 compared to IgG control antibodies (**Figure 3A**). IT-901 and LPS respectively reduced and enhanced the binding of p65 to the promoters, and IT-901 impaired the LPS-dependent enhancement (**Figure 3B**).

In line with the ROS-dependent impairment in NF-κB activity in MEC-1 cells (**Figure 1D**), p65 binding to the *ccr2*, *cxcr3* and *ccr7* promoters was strongly suppressed by H_2_O_2_ treatment (**Figure 3C**), indicating that intracellular ROS affect the transcriptional activity of the p65 subunit of NF-κB and underscoring a prominent role of p65 in the coordinated transcriptional regulation of CCR2, CXCR3 and CCR7 in MEC-1 cells.

### The p66Shc Deficiency-Related Impairment in ROS Production Leads to Enhanced NF-κB Activation in CLL Cells

**Figure 4:**
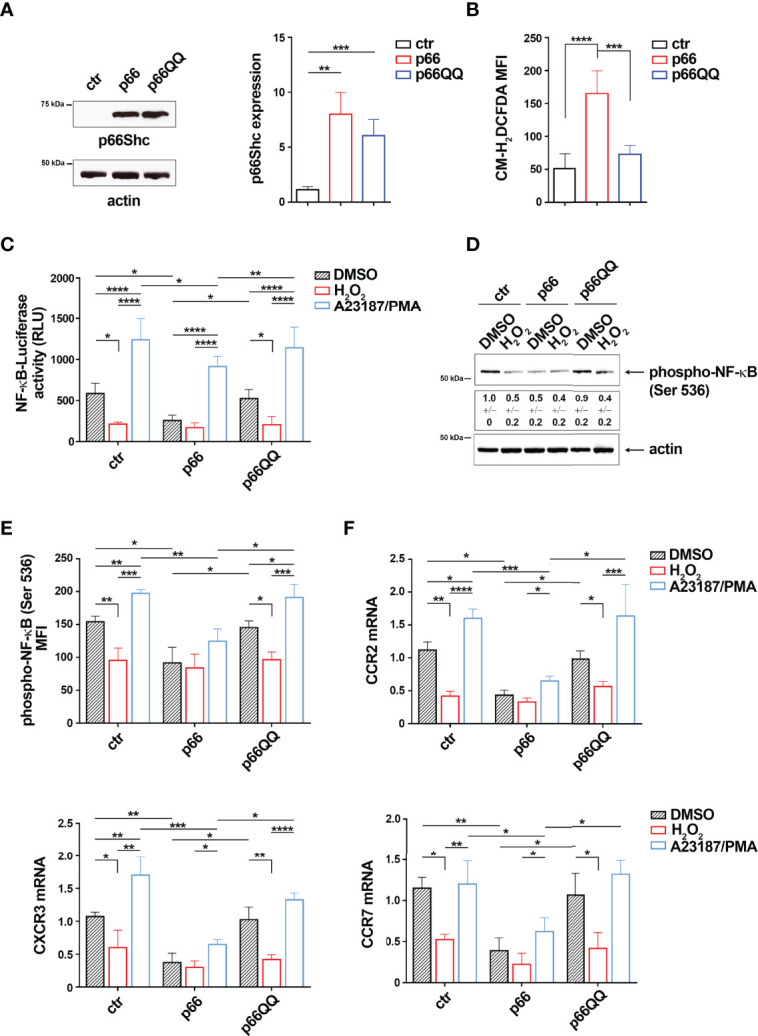
The pro-oxidant activity of p66Shc inhibits NF-κB activity in MEC-1 cells. **(A)** Immunoblot analysis with anti-Shc antibodies of postnuclear supernatants of MEC-1 cells stably transfected with empty vector (ctr) or with a vector encoding either wild-type (p66) or mutated (p66QQ) p66Shc. The stripped filters were reprobed with anti-actin antibodies. Molecular weights (kDa) are indicated on the left of the panel. The quantification of three independent experiments is shown on the right. **(B)** Flow cytometric analysis of ROS intracellular content in MEC-1 transfectants stained with CM-H_2_DCFDA (n independent experiments = 3). **(C)** Quantification of the luciferase activity in MEC-1 stable transfectants transiently transfected with the NF-κB-luciferase reporter construct and then treated for 6 h with either DMSO, or 100 μM H_2_O_2_ or A23187+PMA. Data are expressed as relative luciferase units (RLU) (n independent experiments = 4). **(D, E)** Immunoblot **(D)** and flow cytometric **(E)** analysis of phospho-NF-κB (Ser536) in MEC-1 transfectants, transiently transfected with the NF-κB-luciferase reporter construct and then treated for 30 min as above. The stripped filters were reprobed with anti-actin antibodies. Molecular weights (kDa) are indicated on the left of the panel. [**(D)** n independent experiments = 3; **(E)** n independent experiments = 4]. **(F)** Quantitative RT-PCR analysis of CCR2, CXCR3 and CCR7 mRNA in MEC-1 cells transfected and treated as in **(C)** The relative gene transcript abundance was determined on triplicate samples using the ddCt method and normalized to HPRT1 (n independent experiments = 4). Mean ± SD. C-F. Anova two-way test, Multiple Comparison. **(A, B)**. Mann Whitney Rank Sum Test. p ≤ 0.0001, ****; p ≤ 0.001, ***; p ≤ 0.01, **; p ≤ 0.05, *.

p66Shc is a pro-apoptotic adaptor with ROS-elevating activity (12). Given the inverse relationship between NF-κB activation and intracellular ROS in B cells (**Figure 1**), we addressed the potential role of the pro-oxidant activity of p66Shc in NF-κB activation. To this aim we used MEC-1 cells, which do not express p66Shc due to promoter methylation (3), stably transfected with a ROS-defective mutant carrying E to Q substitutions at positions 132-133 (p66QQ), which disrupt cytochrome-c binding (**Figure 4A**). The empty vector transfectant lacking p66Shc (ctr) and a transfectant expressing the wild-type protein (p66) were used as controls. Flow cytometric analysis of homeostatic ROS production in the CM-H_2_DCFDA-loaded MEC-1 transfectants confirmed an enhanced ROS production in p66Shc-expressing cells, but not in cells expressing p66ShcQQ, compared to control cells (**Figure 4B**).

To evaluate the implication of p66Shc-derived ROS on NF-κB activity, we transiently transfected the MEC-1 transfectants with the NF-κB-luciferase reporter and measured the luciferase activity after 24 h. The basal NF-κB-dependent luciferase activity was strongly decreased in the p66 transfectant compared to the empty vector control transfectant (**Figure 4C**). Moreover NF-κB activation, assessed by flow cytometric and immunoblot analysis of transfectants using anti-phospho-NF-κB p65 (Ser536) antibodies, was significantly impaired in the p66 transfectant compared to the empty vector control transfectant (**Figures 4D, E**; **Supplementary Figure 3B**). Interestingly, treatment of ctr cells with 100 μM H_2_O_2_ strongly reduced both NF-κB phosphorylation and NF-κB-dependent luciferase activity to levels similar to the untreated p66 transfectant (**Figures 4C–E**), with no significant effect on cell viability of transfected cells (**Supplementary Figure 3B**). Expression of the ROS-defective p66Shc mutant reversed the p66Shc-dependent impairment in NF-κB phosphorylation and NF-κB-dependent luciferase activity (**Figures 4C–E**), suggesting that p66Shc inhibits NF-κB through its ROS-elevating activity.

Consistent with their shared NF-κB-dependent regulation, the basal mRNA levels of CCR2, CXCR3 and CCR7 were reduced in the p66 transfectant, but not in the p66QQ transfectant (**Figure 4F**). Of note, H_2_O_2_ did not reduce either NF-κB activity or chemokine receptor expression in the p66Shc transfectant (**Figures 4C–F**), indicating that the endogenous p66Shc-generated ROS were sufficient to elicit the maximal cellular response to ROS elevation. Collectively, these results demonstrate that p66Shc controls the NF-κB-mediated expression of the chemokine receptors CCR2, CXCR3 and CCR7 through its pro-oxidant activity.

### p66Shc Reconstitution in CLL Cells Normalizes NF-κB Activation in a ROS-dependent Manner

**Figure 5:**
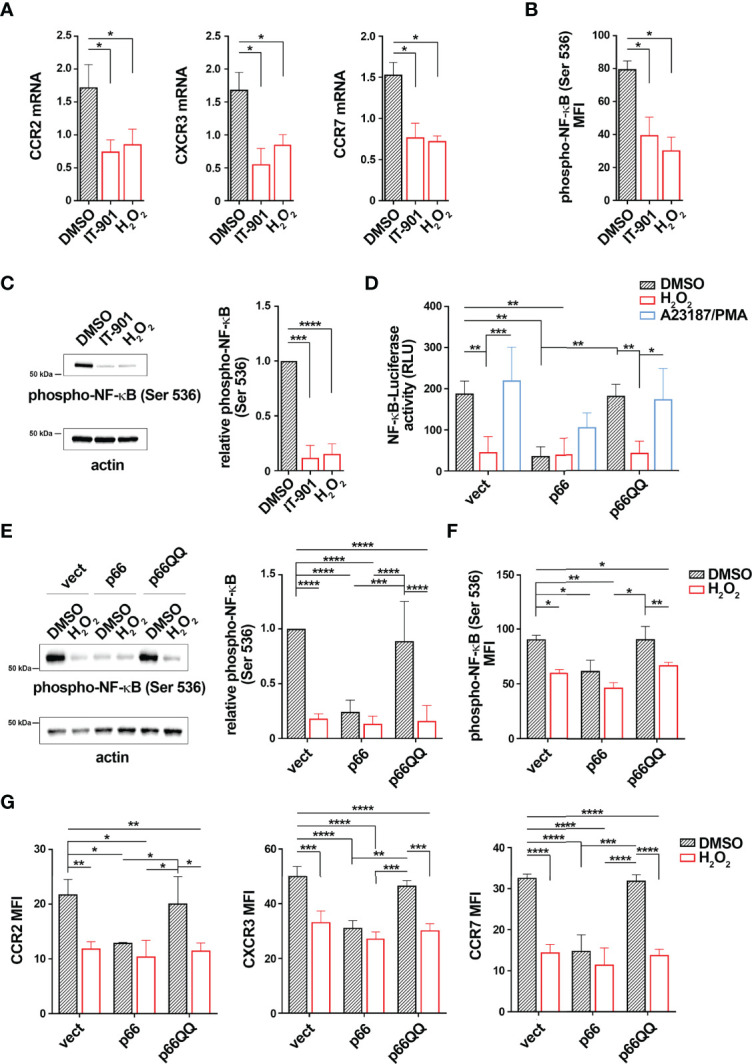
p66Shc normalization inhibits NF-κB activity in CLL cells. **(A)** Quantitative RT-PCR analysis of CCR2, CXCR3 and CCR7 mRNA in B lymphocytes purified from peripheral blood of CLL patients (CLL; n CLL patients = 5) treated for 24 h with either 1 μM IT-901 or 100 μM H_2_O_2_. The relative gene transcript abundance was determined on triplicate samples using the ddCt method and normalized to HPRT1. **(B, C)** Flow cytometric **(B)** and immunoblot **(C)** analysis of phospho-NF-κB (Ser536) in B lymphocytes purified from peripheral blood of CLL patients (CLL) and then treated as above [**(B)** n CLL patients = 5; **(C)** n CLL patients = 3). **(D)**] Quantification of the luciferase activity in B lymphocytes purified from peripheral blood of CLL patients (CLL; n CLL patients = 4) transiently transfected with the NF-κB-luciferase reporter in combination with empty vector (vect) or a vector encoding either wild-type (p66) or mutated (p66QQ) p66Shc. 16 h after transfection cells were treated with either DMSO, or 100 μM H_2_O_2_ or A23187+PMA. Data are expressed as relative luciferase units (RLU). **(E, F)** Immunoblot **(E)** and flow cytometric **(F)** analysis of phospho-NF-κB (Ser536) in B lymphocytes purified from peripheral blood of CLL patients (CLL); then transfected as in **D** and treated for 30 min with either DMSO, or 100 μM H_2_O_2_ or A23187+PMA [**(E)** n CLL patients = 4; **(F)**: n CLL patients = 4]. **(G)** Quantitative RT-PCR analysis of CCR2, CXCR3 and CCR7 mRNA in B lymphocytes purified from peripheral blood of CLL patients (CLL; n CLL patients = 4), transfected and treated as in **(C)** The relative gene transcript abundance was determined on triplicate samples using the ddCt method and normalized to HPRT1. Mean ± SD. D-F. Anova two-way test, Multiple Comparison. **(A, B)**. Mann Whitney Rank Sum Test. **(C)** One-sample Wilcoxon test. p ≤ 0.0001, ****; p ≤ 0.001, ***; p ≤ 0.01, **; p ≤ 0.05, *.

NF-κB activity is potentiated in CLL cells (25). Since CLL cells show an overexpression of CCR2, CXCR3 and CCR7 compared to healthy B cells (4, 7), we asked whether this is caused by the enhanced NF-κB activity. Treatment of CLL cells with the NF-κB inhibitor IT-901 reduced NF-κB phosphorylation and strongly decreased the mRNA levels of CCR2, CXCR3 and CCR7 (**Figures 5A–C**) without significantly affecting cell viability (**Supplementary Figure 4**), supporting the notion that NF-κB activation plays a key role in the coordinated transcriptional control of CCR2, CXCR3 and CCR7 in CLL cells. Of note, similar to IT-901, treatment of CLL cells with H_2_O_2_ as exogenous source of ROS reduced both NF-κB phosphorylation and mRNA expression of these chemokine receptors (**Figures 5A–C**). These results suggest that enhanced intracellular ROS levels counteract NF-κB activation, limiting the expression of chemokine receptors in B cells.

Intracellular ROS are reduced in CLL cells, as a result of the defective expression of p66Shc compared to healthy B cells (**Supplementary Table 1** and **Supplementary Table 4**). We asked whether p66Shc reconstitution in CLL cells hampers NF-κB hyperactivation. Leukemic cells were isolated from CLL patients and transiently nucleofected with the NF-κB luciferase reporter construct. Cells were co-transfected with vectors encoding either wild-type p66Shc (p66) or the ROS defective p66ShcQQ mutant (p66QQ). Cells co-transfected with empty vector were used as control (vect). 16 h after transfection cells were left untreated or treated with 100 μM H_2_O_2_. Cells stimulated with a combination of the calcium ionophore A23187 and the diacylglycerol analogue phorbol myristate acetate (PMA) were used as positive controls (29) (30). NF-κB-dependent luciferase activity and NF-κB phosphorylation were assessed by luciferase assays and flow cytometric analysis of cells stained with a phospho-NF-κB p65-specific antibody, respectively. p66Shc expression, NF-κB luciferase reporter transfection efficiency and intracellular ROS were measured by real-time qPCR and flow cytometric analysis of luciferase^+^ cells and CM-H_2_DCFDA-stained cells, respectively (**Supplementary Figure 1** and **Supplementary Table 5**). Viability of transfectants was assessed by flow cytometric analysis of Annexin V^-^/PI^-^ cells (**Supplementary Figure 5**). In agreement with our previous results (4), reconstitution of CLL cells with wild-type p66Shc, but not with the p66ShcQQ mutant, enhanced intracellular ROS compared to the empty vector control (**Supplementary Figure 5**). CLL cells transfected with empty vector showed high NF-κB phosphorylation and activity, which were attenuated by H_2_O_2_ (**Figures 5D–F**). Conversely, reconstitution of CLL cells with wild-type p66Shc, but not with the p66QQ mutant, elicited an impairment in both NF-κB-dependent luciferase activity and NF-κB phosphorylation under basal conditions comparable to H_2_O_2_ treatment in control cells (**Figures 5D–F**). Our data demonstrate that restoring ROS production by forced p66Shc expression hampers NF-κB hyperactivation in CLL cells.

Since NF-κB is a common transcription factor for CCR2, CXCR3 and CCR7, surface and mRNA expression of the three receptors was next measured in all transfectants. The wild-type p66Shc-expressing transfectant, but not the p66QQ transfectant, showed reduced basal mRNA levels of all three receptors compared to the control transfectant (**Figure 5G**). Treatment with H_2_O_2_ led to a decrease in receptor expression in control and p66ShcQQ-expression cells, but not in cells expressing wild-type p66Shc (**Figure 5G**). Taken together these results demonstrate that the p66Shc-dependent decrease in intracellular ROS contributes to promote NF-κB activity in CLL cells, thereby enhancing the expression of the homing receptors CCR2, CXCR3 and CCR7.

## Discussion

ROS are highly reactive chemical compounds generated by biological reactions. Notwithstanding the fact that in the majority of cases they represent byproducts, when released in specific subcellular locations and at certain concentrations, ROS become key signaling mediators (40). Indeed, ROS control the activity of several ROS-sensitive transcription factors, as clearly exemplified by transcription factors of the forkhead box - class O (FoxO) family, whose activity is regulated by ROS at multiple levels to promote the cellular antioxidant defense (34). NF-κB, a family of 15 protein complexes that, from a pool of 5 monomers, work as hetero- and homo-dimers (41), is also regulated by ROS, although the underlying molecular mechanisms and the effects on NF-κB transcriptional activities are still poorly understood (16). Here we demonstrated that, in the CLL-derived B cell line MEC-1, the exogenous administration of ROS suppresses the transcriptional activity of the p65 subunit of NF-κB in a dose-dependent manner, suggesting that redox signaling negatively controls NF-κB activation in these cells. The remarkable capability of NF-κB to control hundreds of target genes (33, 42) makes ROS an interesting regulatory molecules in the biology of B lymphocytes.

NF-κB is mainly cytoplasmic due to the binding of a dedicated set of inhibitory proteins comprising the “Inhibitor of NF-κB” (IκB) family (43). The intricate biochemical pathways controlling NF-κB/inhibitor complexes, known as “canonical” and “alternative” pathways, lead to post-translational modification of IκB and release of NF-κB dimers that translocate to the nucleus (42). Depending on cell type and on their source, ROS either regulate the phosphorylation or oxidize components of the NF-κB regulatory complex, respectively activating or inhibiting NF-κB. Exogenous ROS affect IκBα degradation by altering its tyrosine phosphorylation pattern (44). On the other hand, treatment of breast cancer cell line 4T1 with sodium selenite, a source of inorganic selenium, causes a transient increase in intracellular ROS levels that in turn inhibits p65 phosphorylation (18). In B lymphocytes, NF-κB transduces a plethora of pro-survival signaling cascades to the nucleus (45). Our finding that NF-κB activity is negatively regulated by ROS in B lymphocytes opens to the possibility that a tight balance of redox signaling is required to control NF-κB-dependent cell survival. However, little information is available to date concerning oxidation of the signaling molecules that control NF-κB activation in B lymphocytes.

Oxidative stress has been linked to several pathologies, spanning from inflammatory diseases to premature ageing and cancer (40). Under pathological conditions, the delicate intracellular ROS balance is perturbed and usually leads to ROS accumulation and oxidative stress (46). In particular, increased intracellular ROS levels have been linked to cancer initiation, progression, neo-angiogenesis, and metastasis (47), making ROS downregulation a promising strategy for controlling the disease. However, levels of ROS spanning from low to moderate also display pro-tumoral activities due to their ability to promote cell survival and proliferation (48). Huang and colleagues recently reported that low levels of ROS drive progression and therapeutic resistance of glioblastoma (49). In these cells a rise in intracellular ROS might potentially be beneficial for tumor suppression by enhancing the expression of tumor suppressor genes, such as *p53* (50), or by activating apoptotic and autophagic processes, eventually leading to tumor cell death (51). Many drugs, including chemo- and radiotherapeutic agents, take advantage of these mechanisms and, by elevating ROS levels, efficiently kill cancer cells (52). We recently reported that CLL cells have a low intracellular ROS content as a consequence of defective expression of the adaptor protein p66Shc, which correlates to CLL pathogenesis (4). The low intracellular content of ROS is related to the enhanced expression of the homing receptors CCR2, CXCR3 and CCR7, which promote both entry and accumulation of CLL cells in the pro-survival tumor microenvironment of lymphoid organs, eventually enhancing leukemic cell survival (4, 5, 7, 8). Here we added a tile to the puzzle by demonstrating that the low intracellular content of ROS contributes to promote the activation of the p65 subunit of NF-κB in CLL cells, which in turn transcriptionally enhances the expression of the homing receptors CCR2, CXCR3 and CCR7. Of note, forced expression of p66Shc in CLL cells leads to an increase in intracellular ROS, thereby suppressing NF-κB activation and normalizing the expression of these homing receptors.

Aberrant activation of the NF-κB pathway in leukemic cells from CLL patients plays a major role in disease development and evolution (25). Only a few recurrently mutated NF-κB-related genes have been identified in CLL (i.e. *BIRC3*, *MYD88* and *NFKBIE* mutations) and often at a low frequency (53) (54) (55). Rather, aberrant NF-κB activation relies on the tumor microenvironment, which represents a source of stimuli for a number of surface receptors, including the BCR, TLRs and CD40 (5, 22, 23, 56). The importance of cell-extrinsic triggering for CLL pathophysiology was recently also highlighted by the clinical efficacy of novel drugs targeting microenvironmental interactions through the inhibition of BCR signaling, e.g., Acalabrutinib and Duvelisib (57). Therefore, a tight regulation of NF-κB activation is of fundamental importance for the therapeutic management of CLL. We now propose that low intracellular ROS levels contribute to aberrant NF-κB activation in CLL. The tight control of intracellular ROS appears therefore as a promising therapeutic strategy to overcome aberrant NF-κB activation. It is however noteworthy that the high reactivity of ROS makes them harmful to protein, DNA, and lipids, as witnessed by their tight control by antioxidant systems that maintain ROS homeostasis (58). Direct pharmacological modulation of ROS-modulating enzymes might therefore lead to uncontrolled side effects that make this approach hardly feasible (59). Here we demonstrated that reconstitution of the pro-oxidant adaptor p66Shc, whose expression is profoundly impaired in CLL cells (2), enhances intracellular ROS, partly suppressing NF-κB aberrant activation. The subsequent normalization of homing receptors might counteract the accumulation of CLL in the pro-survival and protective niche of lymphoid organs, finally making them more susceptible to therapies.

Based on our findings, p66Shc reconstitution appears as a promising future strategy to control NF-κB activity and at least in part counteract disease progression. We have previously reported that p66Shc expression is positively regulated by the transcription factor STAT4, whose expression is also profoundly defective in CLL cells (3). This suggests that pharmacological compounds designed to either enhance STAT4 expression or promote activation of the residual STAT4 might represent interesting future developments for the treatment of this as yet uncurable disease.

## Data Availability Statement

The original contributions presented in the study are included in the article/**Supplementary Material**. Further inquiries can be directed to the corresponding authors.

## Ethics Statement

The studies involving human participants were reviewed and approved by Local Ethics Committees of the University of Siena and of the University of Padua. The patients/participants provided their written informed consent to participate in this study.

## Author Contributions

VT, GB, NC, NM, FF, AV, LT, LP, and CB designed research and analyzed and interpreted data; VT, GB, NC, NM, FF, and AV performed research; LT contributed vital reagents; VT, GB, NC, FF, AV, LT, LP, and CB drafted the manuscript. All authors contributed to the article and approved the submitted version.

## Funding

The research leading to these results has received funding from AIRC under IG 2017 - ID. 20148 –, Regione Toscana, ID. PRECISE-CLL- and EU (ERC-2021-SyG 951329) to CB. This work was also supported by a grant from AIRC to LT (IG-25024).

## Conflict of Interest

The authors declare that the research was conducted in the absence of any commercial or financial relationships that could be construed as a potential conflict of interest.

## Publisher’s Note

All claims expressed in this article are solely those of the authors and do not necessarily represent those of their affiliated organizations, or those of the publisher, the editors and the reviewers. Any product that may be evaluated in this article, or claim that may be made by its manufacturer, is not guaranteed or endorsed by the publisher.
