# Supplementary material for: p66Shc Deficiency in Chronic Lymphocytic Leukemia Promotes Chemokine Receptor Expression Through the ROS-Dependent Inhibition of NF-κB
Source: Front Oncol. 2022 Jun 29;12:877495. doi: 10.3389/fonc.2022.877495 (PMC9278989; doi:10.3389/fonc.2022.877495)
Supplement: Supplementary file 1 [file DataSheet_1.pdf]

*Supplementary Material*

## 1. Supplementary Figures

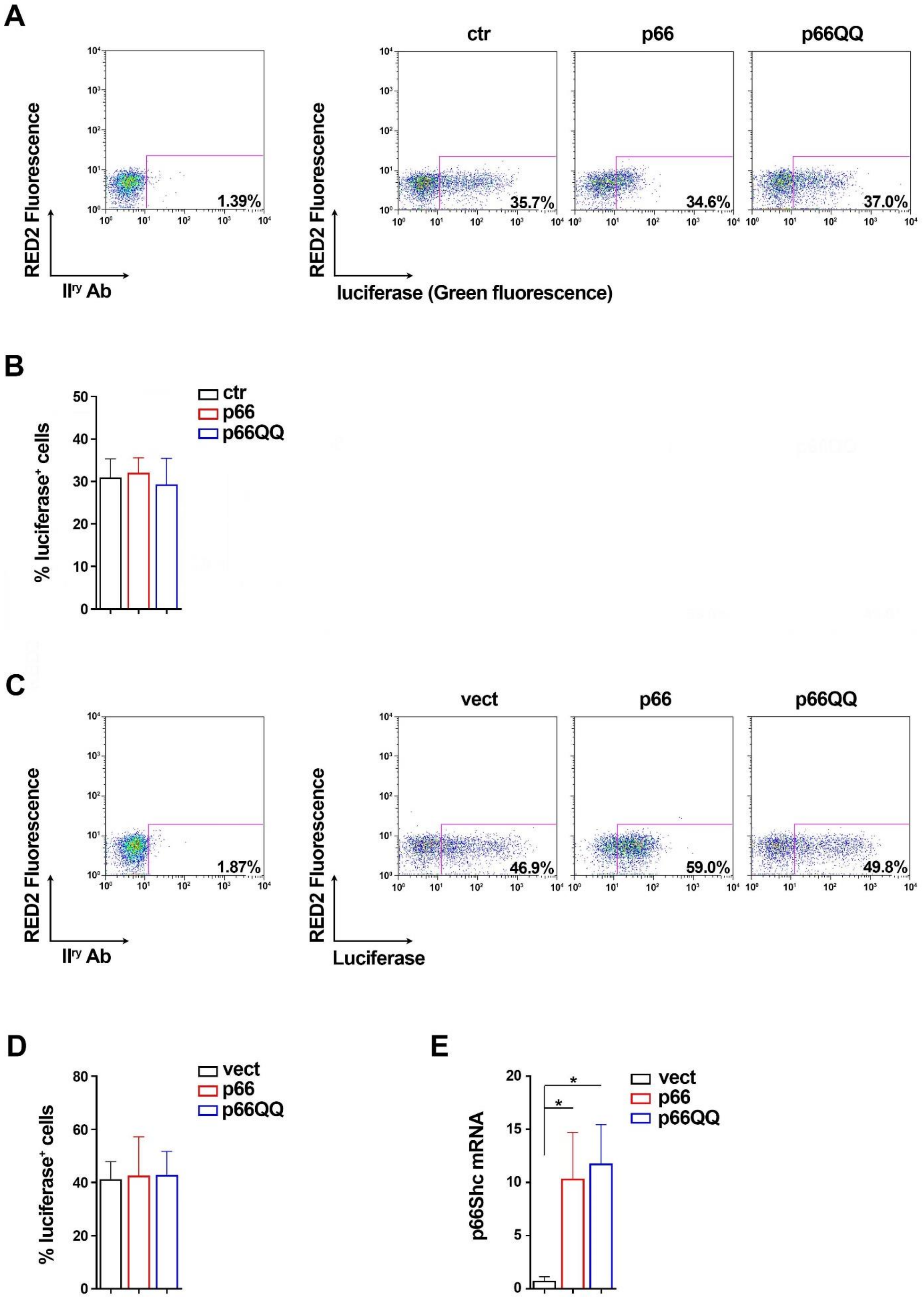
A
ctr
p66
p66QQ
RED2 Fluorescence
Il^ry Ab
1.39%
35.7%
34.6%
37.0%
luciferase (Green fluorescence)
B
ctr
p66
p66QQ
% luciferase+ cells
C
vect
p66
p66QQ
1.87%
46.9%
59.0%
49.8%
Luciferase
D
vect
p66
p66QQ
% luciferase+ cells
E
vect
p66
p66QQ
p66Shc mRNA

**Supplementary Figure 1.**

(**A-D**). Flow cytometric analysis of the percentage of luciferase$^{+}$ cells in MEC-1 transfectants (**A**, **B**) or in B lymphocytes purified from peripheral blood of CLL patients (CLL; n=3) (**C**, **D**), transiently transfected with the NF-κB-luciferase construct alone (**A**, **B**) or in combination with empty vector (vect) or a vector encoding either wild-type (p66) or mutated (p66QQ) p66Shc (**C**, **D**). **E**. Quantitative RT-PCR analysis of p66Shc mRNA in B lymphocytes purified from peripheral blood of CLL patients (CLL; n=4) transiently transfected with the NF-κB-luciferase reporter in combination with empty vector (vect) or a vector encoding either wild-type (p66) or mutated (p66QQ) p66Shc. The relative gene transcript abundance was determined on triplicate samples using the ddCt method and normalized to HPRT1. Anova two-way test, Multiple Comparison. $p \leq 0.05$, *.

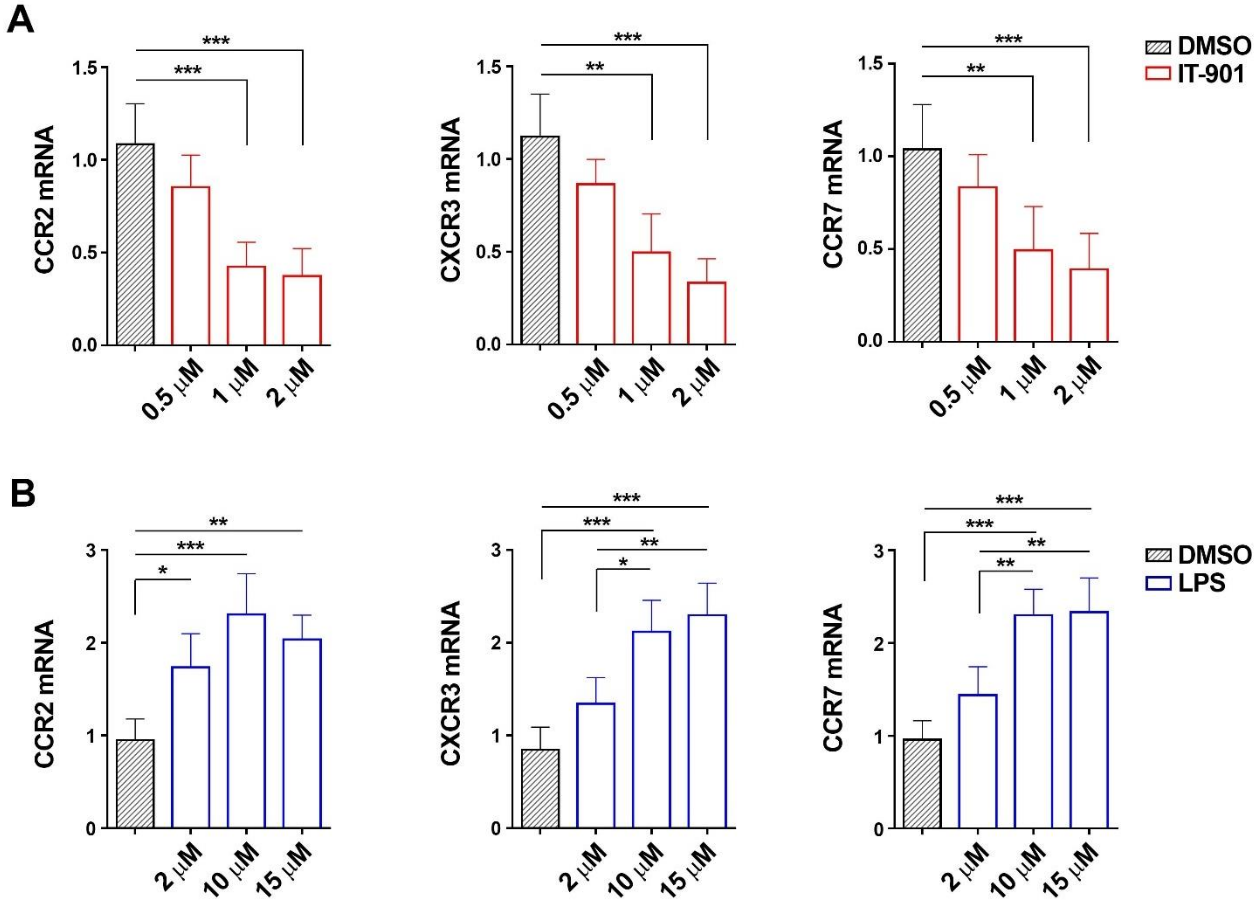


**Supplementary Figure 2.**

(**A, B**). Quantitative RT-PCR analysis of CCR2, CXCR3 and CCR7 mRNA in MEC cells treated with IT-901 (**A**) or LPS (**B**) at the indicated concentrations for 24 h at 37°C. The relative gene transcript abundance was determined on triplicate samples using the ddCt method and normalized to HPRT1 (n=3). Mean±SD. Anova two-way test, Multiple Comparison. $p \le 0.001$, ***; $p \le 0.01$, **; $p \le 0.05$, *.

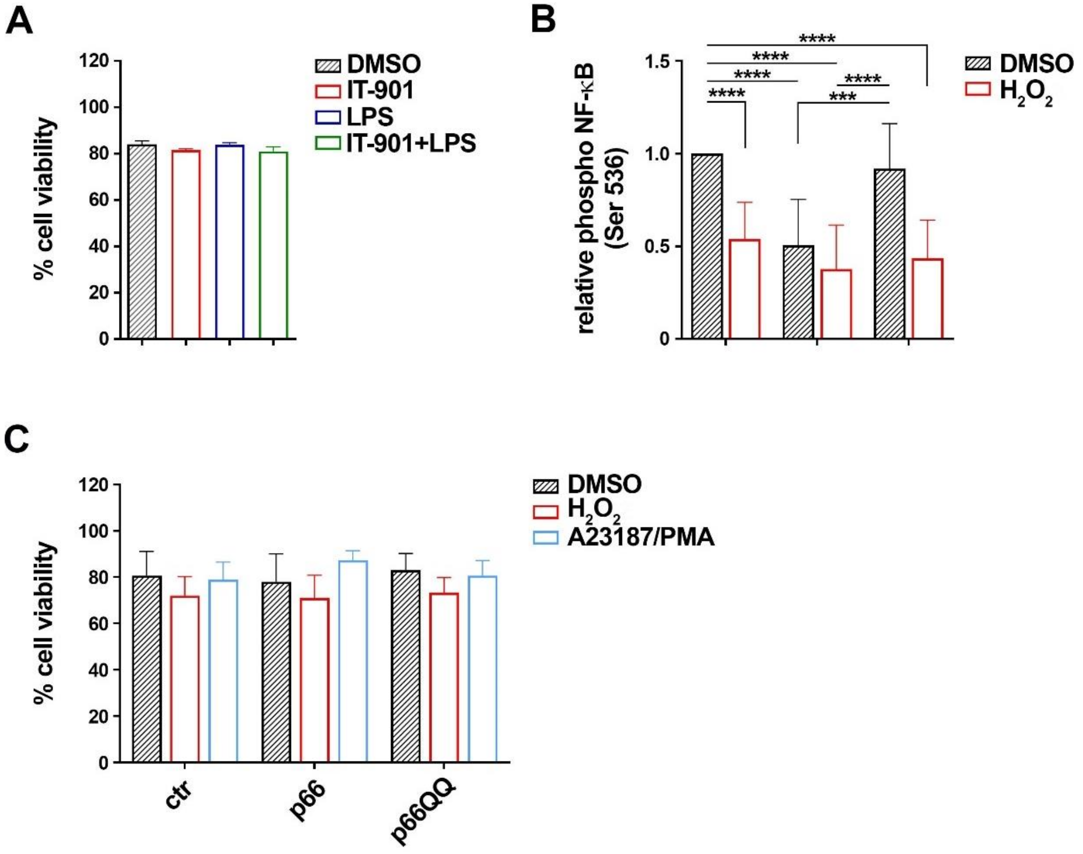


**Supplementary Figure 3.**

(**A**) Flow cytometric analysis of cell viability in MEC-1 cells treated with 1 μM IT-901 or 10 μM LPS or the combination of both for 24 h at 37°C. Cell viability was calculated as the percentage of Annexin $V^-/PI^-$ cells (n independent experiments=4). (**B**) Immunoblot analysis of phospho-NF-κB (Ser536) in MEC-1 transfectants, transiently transfected with the NF-κB-luciferase reporter construct and then treated for 30 min with either DMSO or 100 μM $H_2O_2$. The stripped filters were reprobed with anti-actin antibodies. The quantification of eight independent experiments is shown. (**C**) Flow cytometric analysis of cell viability in MEC-1 stable transfectants transiently transfected with the NF-κB-luciferase reporter construct and then treated for 24 h with either DMSO, or 100 μM $H_2O_2$ or A23187+PMA. Cell viability was calculated as the percentage of Annexin $V^-/PI^-$ cells (n independent experiments=3). Mean±SD. Anova two-way test, Multiple Comparison. $p \leq 0.0001$, ****; $p \leq 0.001$, ***.

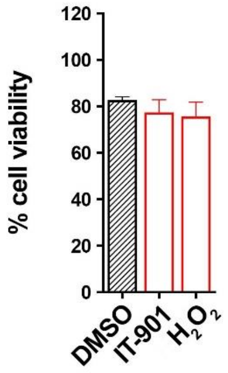


**Supplementary Figure 4.**

Flow cytometric analysis of cell viability in CLL cells treated with 1 μM IT-901 or 100 μM $H_2O_2$ for 24 h at 37°C. Cell viability was calculated as the percentage of Annexin $V^-$/$PI^-$ cells (n independent experiments = 6). Mean±SD. Anova two-way test, Multiple Comparison.

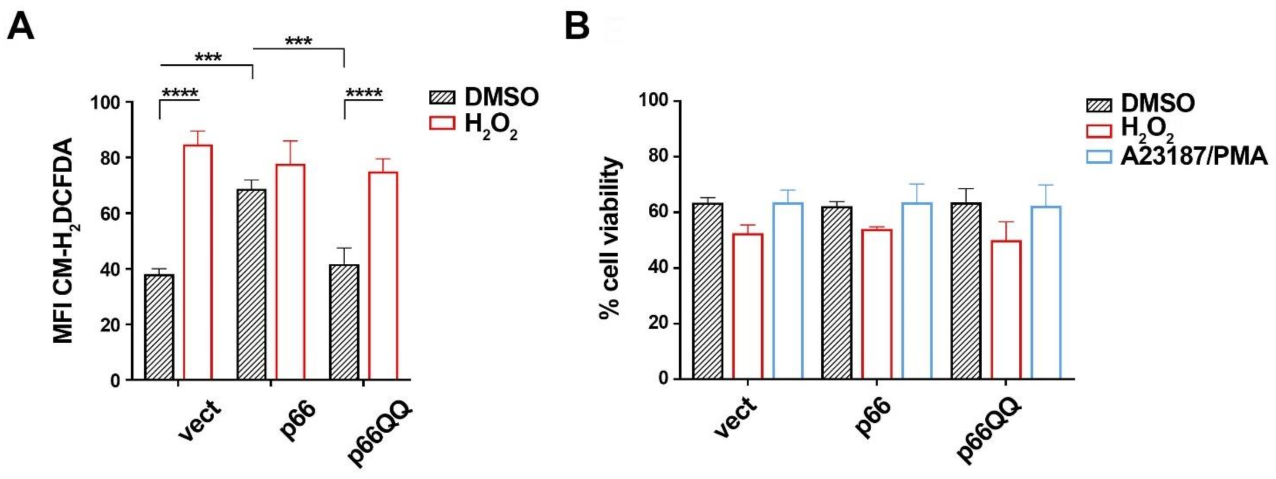


**Supplementary Figure 5.**

(**A**). Flow cytometric analysis of ROS intracellular content in cells described and treated as in (**A**) and stained with 5 μM CM-$H_2$DCFDA. (**B**). Flow cytometric analysis of cell viability in CLL cells transiently transfected with the NF-κB-luciferase reporter construct and then treated for 24 h with either DMSO, or 100 μM $H_2O_2$ or A23187+PMA. Cell viability was calculated as the percentage of Annexin $V^-/PI^-$ cells (n independent experiments=3). Mean±SD. Anova two-way test, Multiple Comparison. p≤0.0001, ****; p≤0.001, ***; p≤0.01, **; p≤0.05, *.

## 2. Supplementary Tables

**Supplementary Table 1. Clinical parameters of CLL patients used in this study.** IGHV: Immunoglobulin heavy variable chain; WBC: white blood cell count; Ly: lymphocytes; HD: healthy donor.

| SAMPLE | MUTATIONAL IGHV STATUS | KARYOTYPE | WBC (n/ml) | LY (%) | p66Shc mRNA (ΔΔCT) |
|---|---|---|---|---|---|
| **CLL # 1** | mutated | normal | 17.590 | 65.2 | 0.28 |
| **CLL # 2** | mutated | 13q | 24.730 | 79.6 | 0.43 |
| **CLL # 3** | mutated | 13q | 12.900 | 66.2 | 0.50 |
| **CLL # 4** | mutated | 13q | 52.810 | nd | 0.38 |
| **CLL # 5** | mutated | normal | 16.380 | 72.5 | 0.20 |
| **CLL # 6** | mutated | 13q | 10.770 | 67.8 | 0.60 |
| **CLL # 7** | mutated | 11q | 9.210 | 92.0 | 0.68 |
| **CLL # 8** | mutated | 12+ | 83.750 | 69.2 | 0.48 |
| **CLL # 9** | unmutated | 13q | 47.830 | 97.0 | 0.02 |
| **CLL # 10** | mutated | 11q | 27.120 | 69.4 | 0.20 |
| **CLL # 11** | unmutated | 17p | 39.420 | 91.3 | 0.18 |
| **CLL # 12** | mutated | 13q | 32.960 | 93.0 | 0.45 |
| **CLL # 13** | unmutated | 11q | 69.100 | 81.6 | 0.01 |
| **CLL # 14** | unmutated | 17p | 19.900 | 70.3 | 0.16 |
| **CLL # 15** | unmutated | normal | 113.700 | 96.6 | 0.08 |
| **CLL # 16** | unmutated | 12+ | 35.190 | 73.6 | 0.21 |
| **CLL # 17** | mutated | 13q | 22.450 | 77.7 | 0.34 |
| **CLL # 18** | unmutated | 13q | 206.800 | 98.5 | 0.05 |
| **CLL # 19** | unmutated | 12+ | 65.700 | 86.2 | 0.02 |
| **CLL # 20** | mutated | 13q | 30.000 | 83.7 | 0.38 |
| **CLL # 21** | mutated | 13q | 66.400 | 88.7 | 0,12 |
| **CLL # 22** | unmutated | 12+ | 66.900 | 93.3 | 0,09 |
| **CLL # 23** | mutated | 13q | 88.230 | 90.9 | 0,30 |
| **CLL # 24** | unmutated | 11q | 124.900 | 95.9 | 0.09 |
| **CLL # 25** | mutated | normal | 26.680 | 83.9 | 0.29 |
| **CLL # 26** | mutated | 13q | 15.430 | 81.4 | 0.13 |
| **CLL # 27** | mutated | normal | 43.600 | 82.6 | 0.01 |
| **CLL # 28** | unmutated | 17p | 23.600 | 67.8 | 0.03 |
| **CLL # 29** | mutated | normal | 69.740 | 94.0 | 0.26 |
| **HD #1** | - | - | - | - | 1.24 |
| **HD #2** | - | - | - | - | 1.00 |
| **HD #3** | - | - | - | - | 1.67 |
| **HD #4** | - | - | - | - | 1.31 |
| **HD #5** | - | - | - | - | 0.92 |
| **HD #6** | - | - | - | - | 1.36 |
| **HD #7** | - | - | - | - | 1.03 |

| | | | | | |
|---|---|---|---|---|---|
| **HD #8** | - | - | - | - | 1.58 |
| **HD #9** | - | - | - | - | 0.91 |
| **HD #10** | - | - | - | - | 0.99 |
| **HD #11** | - | - | - | - | 1.23 |
| **HD #12** | - | - | - | - | 1.01 |
| **HD #13** | - | - | - | - | 1.10 |
| **HD #14** | - | - | - | - | 1.89 |

**Supplementary Table 2. List of the primers used in this study.**

| | PRIMER FORWARD | PRIMER REVERSE |
|---|---|---|
| **CCR2** | ATGGTCATCTGCTACTCGGG | AGGCCGAATTCCTGGAA |
| **CCR7** | GGAGACTTCTTGGCTTGGTGAG | CCCGCTGGCTTGGAGGAC |
| **CXCR3** | GGATGTGGATGCTGCTCTTG | CGAGGATATTGGGGAGAGCC |
| **P66SHC** | TCCGGAATGAGTCTCTGTCA | GAAGGAGCACAGGGTAGTGG |
| **HPRT1** | AGATGGTCAAGGTCGCAAG | GTATTCATTATAGTCAAGGGCATATC |
| **CCR2 A (CHIP)** | ACTTGCCAAGTCCACAGCAT | TCTCTTGTGTCAGGAACGTCC |
| **CCR2 B (CHIP)** | AGTCTGATGTCCCCATTGCA | TTGTCTATCTGTGTGTGGCTT |
| **CXCR3 C (CHIP)** | AAATGGACTTGCCTCCCAGG | GGCTGGGGCTGAGACTATCT |
| **CXCR3 D (CHIP)** | TGGTCAAGGGTTTGGCTCAG | GGTTGCTCTGGAGACTGCAA |
| **CCR7 E (CHIP)** | GGCTGGCAGATCACCTTAGG | TACTCTGTTGCACAGGCTGG |
| **CCR7 F (CHIP)** | TGGATCCATACCACCACATAGG | ACTCCCTCTCAAGAACCCTGT |

**Supplementary Table 3. List of putative binding sites for NF-κB1 and Rel A (p65) in the promoters of *ccr2, cxcr3* and *ccr7* genes.** TF; transcription factor; NT: nucleotide.

| TF NAME | GENE | SEQUENCE NUMBER | SCORE | START NT | END NT | STRAND | PREDICTED SEQUENCE |
|---|---|---|---|---|---|---|---|
| ***REL* A (p65)** | ***ccr2*** | # 1 | 8.60 | -1043 | -1034 | - | GGAGCATTCC |
| | | # 2 | 7.21 | 376 | 385 | + | TGGAAATGCC |
| | | # 3 | 6.31 | -912 | -903 | + | AGGACGTTCC |
| | | # 4 | 6.10 | -939 | -930 | + | TAGCATTTCC |
| | ***cxcr3*** | # 1 | 10.88 | -2046 | -2037 | - | CTGAATTTCC |
| | | # 2 | 9.66 | -1257 | -1248 | - | GGAACTTTCC |
| | | # 3 | 8.62 | -991 | -982 | - | CTGGTTTTCC |
| | | # 4 | 8.53 | -2231 | -2222 | - | GTGAGTTCCC |
| | | # 5 | 8.24 | -2147 | -2138 | + | TGGACTTGCC |
| | | # 6 | 7.88 | -2047 | -2038 | + | GGGAAATTCA |
| | ***ccr7*** | # 1 | 9.07 | -1492 | -1483 | + | GGGGCTTTCG |
| | | # 2 | 8.14 | -759 | -750 | - | CGGGGTTTCA |
| ***NF-κB1 (p50)*** | ***ccr2*** | # 1 | 8.00 | -664 | -655 | - | GGAGCATTCC |
| | | # 2 | 7.22 | -768 | -759 | + | AGGACGTTCC |
| | ***cxcr3*** | # 1 | 10.63 | -1133 | -1124 | - | GGAACTTTCC |
| | | # 2 | 8.71 | -343 | -334 | + | GGGAAATTCA |
| | | # 3 | 8.47 | -1133 | -1124 | + | GGAAAGTTCC |
| | | # 4 | 8.04 | -159 | -150 | + | GGGAACTCAC |
| | | # 5 | 7.71 | -294 | -285 | - | GGGACTGCCT |
| | | # 6 | 7.20 | -159 | -150 | - | GTGAGTTCCC |
| | | # 7 | 6.99 | -180 | -171 | + | GGGGCTGTCT |
| | | # 8 | 6.95 | -295 | -286 | - | GGGGACTGCC |
| | | # 9 | 6.91 | -665 | -656 | + | GGGGCCCTCC |
| | | # 10 | 6.88 | -160 | -151 | - | GGTGAGTTCC |
| | ***ccr7*** | # 1 | 9.01 | -456 | -447 | - | GGGGTTTCAC |
| | | # 2 | 7.50 | -1150 | -1140 | - | TAGATTTTCCT |
| | | # 3 | 9.08 | -1190 | -1181 | + | GGGGCTTTCG |
| | | # 4 | 8.70 | -1549 | -1539 | - | TGGCTTTCCCT |

**Supplementary Table 4. List of putative DNA binding regions of p65 in the promoters of *ccr2*, *cxcr3* and *ccr7*, amplified by qRT-PCR.** NT: nucleotide.

| DNA BINDING REGION | GENE | START NT | END NT | #PREDICTED SEQUENCE CONTAINED |
|---|---|---|---|---|
| **A** | *ccr2* | -1146 | -1127 | 1 |
| **B** | *ccr2* | -1032 | -811 | 2, 3 |
| **C** | *cxcr3* | -2150 | -1928 | 1, 5, 6 |
| **D** | *cxcr3* | -1373 | -1161 | 2 |
| **E** | *ccr7* | -1544 | -1325 | 1 |
| **F** | *ccr7* | -903 | -691 | 2 |
